# Supplementary material for: Changes in Healthy Behaviors and Meeting 24-h Movement Guidelines in Spanish and Brazilian Preschoolers, Children and Adolescents during the COVID-19 Lockdown
Source: Children (Basel). 2021 Jan 26;8(2):83. doi: 10.3390/children8020083 (PMC7912043; doi:10.3390/children8020083)
Supplement: Supplementary file 1 [file children-08-00083-s001.pdf]

Table S1. Changes in physical activity, screen time and sleep duration before and during the COVID-19 lockdown in both Spanish and Brazil samples.

Variables	Spain				Brazil			
	Pre-lockdown	During lockdown	Dif.	p	Pre-lockdown	During lockdown	Dif.	p
Model 1								
**Preschoolers**								
PA (weekly days)	6.3 (0.1)	5.4 (0.2)	-0.8 (-1.2, -0.4)	<0.001	5.8 (0.2)	4.7 (0.3)	-1.2 (-1.6, -0.8)	<0.001
ST (daily hours)	3.7 (0.3)	5.2 (0.4)	-1.5 (-1.7, -1.2)	<0.001	1.6 (0.2)	2.1 (0.3)	-0.5 (-0.6, -0.3)	<0.001
Sleep (daily hours)	10.3 (0.1)	9.9 (0.2)	-0.4 (-0.8, -0.1)	0.027	10.4 (0.1)	10.6 (0.1)	0.2 (-0.1, 0.5)	0.173
**Children**								
PA (weekly days)	5.4 (0.1)	4.6 (0.2)	-0.8 (-1.1, -0.6)	<0.001	5.3 (0.1)	4.2 (0.2)	-1.1 (-1.3, -0.8)	<0.001
ST (daily hours)	5.3 (0.2)	7.3 (0.3)	-2.1 (-2.2, -1.9)	<0.001	3.9 (0.2)	4.9 (0.3)	-1.1 (-1.2, -1.0)	<0.001
Sleep (daily hours)	10.1 (0.1)	10.0 (0.1)	-0.1 (-0.1, 0.3)	0.221	9.9 (0.1)	10.2 (0.1)	0.3 (0.2, 0.5)	<0.001
**Adolescents**								
PA (weekly days)	4.4 (0.1)	3.8 (0.1)	-0.5 (-0.7, -0.3)	<0.001	4.0 (0.1)	3.0 (0.2)	-1.0 (-1.3, -0.7)	<0.001
ST (daily hours)	8.0 (0.2)	11.1 (0.3)	-3.1 (-3.3, -2.9)	<0.001	6.5 (16.4)	8.3 (0.5)	-1.8 (-2.0, -1.6)	<0.001
Sleep (daily hours)	8.5 (0.1)	9.6 (0.1)	1.1 (0.9, 1.4)	<0.001	8.9 (0.1)	9.5 (0.1)	0.6 (-0.9, -0.4)	<0.001
Model 2								
**Total sample**								
PA (weekly days)	5.0 (0.1)	4.3 (0.1)	-0.7 (-0.8, -0,5)	<0.001	4.9 (0.1)	3.8 (0.1)	-1.1 (-1.2, -0.8)	<0.001
ST (daily hours)	6.5 (0.1)	9.1 (0.2)	-2.5 (-2.6, -2.4)	<0.001	4.5 (0.2)	5.8 (0.2)	-1.3 (-1.4, -1.2)	<0.001
Sleep (daily hours)	9.3 (0.1)	9.8 (0.1)	0.5 (0.4, 0.7)	<0.001	9.6 (0.1)	10.0 (0.1)	0.4 (0.3, 0.5)	<0.001

Data expressed as mean (standard error) or mean differences (95% confident intervals). PA: Physical activity; ST: Screen time. Model 1: Adjusted by sex, breadwinner's educational level, socioeconomic status, BMI (z-score); Model 2: Adjusted by Model 1 + age.

Table S2. Changes in the number (proportion) of the sample meeting the 24-h Movement Guideline recommendations before and during the COVID-19 lockdown by country.

Variables	Spain			Brazil		
	Pre-lockdown	During lockdown	p	Pre-lockdown	During lockdown	p
**Preschoolers**						
PA recommendation	52 (67.5)	37 (48.1)	0.001	36 (50.7)	24 (33.8)	0.002
ST recommendation	4 (5.3)	3 (4.0)	1.000	3 (4.2)	1 (1.4)	0.500
Sleep recommendation	56 (74.7)	44 (58.7)	0.081	47 (66.2)	54 (76.1)	0.167
Meeting 24-h guidelines	4 (5.3)	1 (1.3)	0.375	20 (28.2)	11 (15.5)	0.035
**Children**						
PA recommendation	95 (45.7)	73 (35.1)	0.001	87 (36.1)	65 (27.0)	0.003
ST recommendation	22 (10.6)	9 (4.3)	<0.001	7 (3.0)	4 (1.7)	0.250
Sleep recommendation	200 (96.2)	175 (84.1)	<0.001	207 (87.7)	221 (93.6)	0.016
Meeting 24-h guidelines	13 (6.3)	N/A	N/A	36 (15.3)	24 (10.2)	0.036
**Adolescents**						
PA recommendation	66 (16.4)	54 (16.4)	0.096	30 (15.6)	20 (10.4)	0.076
ST recommendation	13 (4.0)	8 (2.5)	0.063	7 (3.7)	6 (3.2)	1.000
Sleep recommendation	254 (79.1)	293 (91.3)	<0.001	145 (77.1)	159 (84.6)	0.044
Meeting 24-h guidelines	1 (0.3)	1 (0.3)	1.000	2 (1.1)	2 (1.1)	1.000
**Total sample**						
PA recommendation	209 (34.6)	160 (26.5)	<0.001	151 (30.5)	108 (21.8)	<0.001
ST recommendation	30 (5.0)	11 (1.8)	<0.001	124 (25.1)	105 (21.2)	<0.001
Sleep recommendation	510 (84.4)	512 (84.8)	0.937	399 (80.6)	434 (87.7)	<0.001
Meeting 24-h guidelines	18 (3.0)	2 (0.3)	<0.001	58 (11.7)	37 (7.5)	0.002

N/A: Not available.
